# Supplementary material for: Comprehensive Annotation and Functional Exploration of MicroRNAs in Lettuce
Source: Front Plant Sci. 2021 Dec 24;12:781836. doi: 10.3389/fpls.2021.781836 (PMC8739914; doi:10.3389/fpls.2021.781836)
Supplement: Supplementary file 2 [file Data_Sheet_8.ZIP › GSM2230750.plot/Lsa-miR397_Lsat_1_v5_gn_7_89801.1_594_TPlot.pdf]

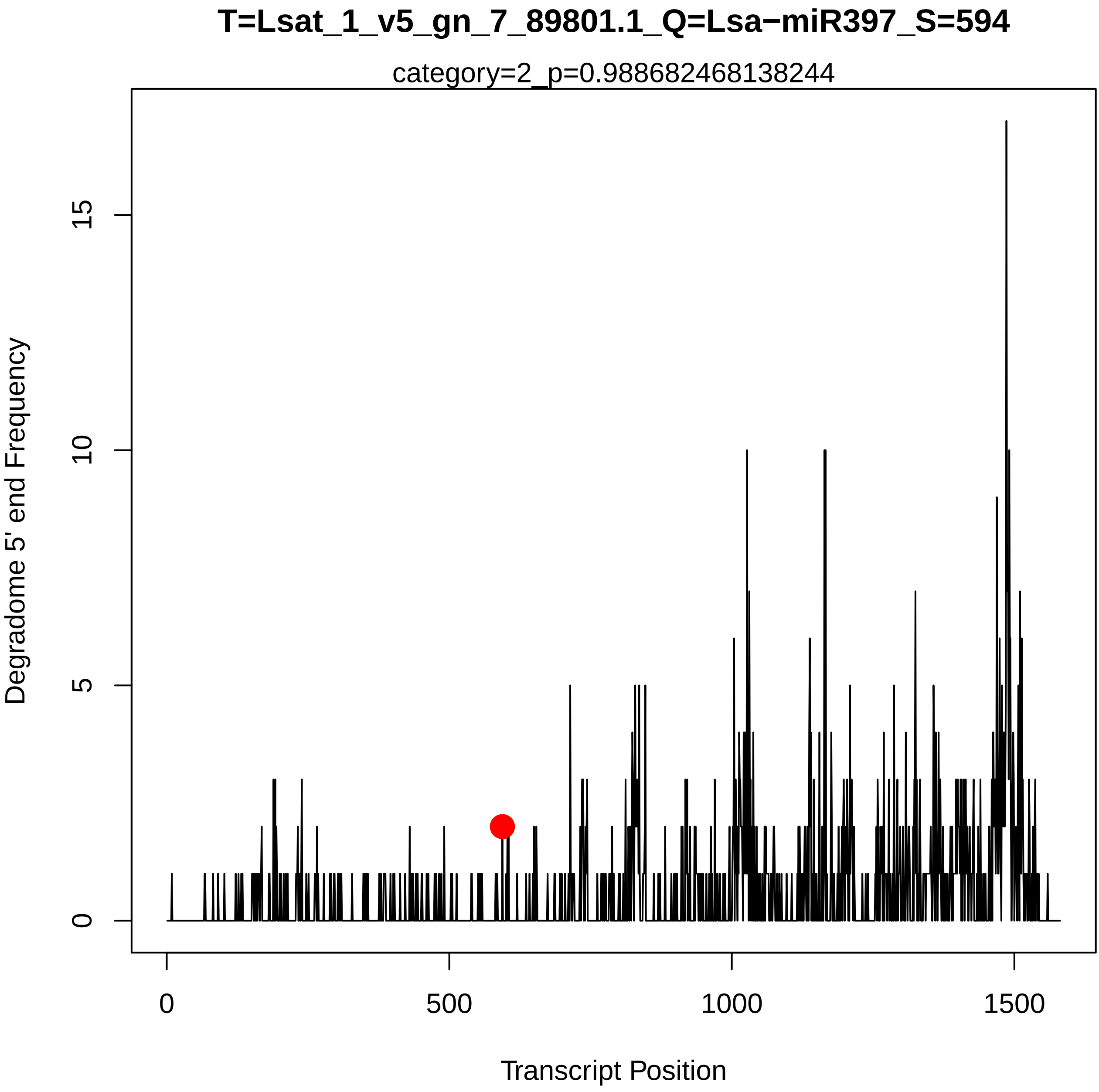

T=Lsat_1_v5_gn_7_89801.1_Q=Lsa−miR397_S=594
category=2_p=0.988682468138244
Degradome 5' end Frequency
Transcript Position
0
5
10
15
0
500
1000
1500
